# Requirement of ATM-dependent pathway for the repair of a subset of DNA double strand breaks created by restriction endonucleases

**DOI:** 10.1186/2041-9414-1-4

**Published:** 2010-05-26

**Authors:** Keiji Suzuki, Maiko Takahashi, Yasuyoshi Oka, Motohiro Yamauchi, Masatoshi Suzuki, Shunichi Yamashita

**Affiliations:** 1 Atomic Bomb Disease Institute, Nagasaki University Graduate School of Biomedical Sciences, 1-12-4 Sakamoto, Nagasaki 852-8523, Japan

## Abstract

**Background:**

DNA double strand breaks induced by DNA damaging agents, such ionizing radiation, are repaired by multiple DNA repair pathways including non-homologous end-joining (NHEJ) repair and homologous recombination (HR) repair. ATM-dependent DNA damage checkpoint regulates a part of DNA repair pathways, however, the exact role of ATM activity remains to be elucidated. In order to define the molecular structure of DNA double strand breaks requiring ATM activity we examined repair of DNA double strand breaks induced by different restriction endonucleases in normal human diploid cells treated with or without ATM-specific inhibitor.

**Results:**

Synchronized G1 cells were treated with various restriction endonucleases. DNA double strand breaks were detected by the foci of phosphorylated ATM at serine 1981 and 53BP1. DNA damage was detectable 2 hours after the treatment, and the number of foci decreased thereafter. Repair of the 3'-protruding ends created by *Pst *I and *Sph *I was efficient irrespective of ATM function, whereas the repair of a part of the blunt ends caused by *Pvu *II and *Rsa *I, and 5'-protruding ends created by *Eco *RI and *Bam *HI, respectively, were compromised by ATM inhibition.

**Conclusions:**

Our results indicate that ATM-dependent pathway plays a pivotal role in the repair of a subset of DNA double strand breaks with specific end structures.

## Background

Ionizing radiation induces various types of DNA damage, among which DNA double strand breaks show the most detrimental effects on living cells. DNA double strand breaks are repaired by two major DNA repair pathways, which are non-homologous end-joining (NHEJ) and homologous recombination (HR) [1-6]. While DNA repair pathway efficiently rejoin the broken ends, un-rejoined or mis-rejoined DNA damage provide chances to threaten the integrity of the genome [7-9]. Thus, the cells evolved a sophisticated system, by which stability of the genome is maintained [10,11]. The system referred to as DNA damage checkpoint pathway requires ATM function [12-14], which is activated by dissociation of ATM proteins followed by autophosphorylation [15]. Activated ATM phosphorylates various downstream proteins including those that regulate cell cycle progression, cell death, as well as DNA repair [11,14,16,17]. Thereby, ATM plays a critical role in orchestrating DNA damage signaling and DNA damage repair.

Although AT cells were known to be sensitive to ionizing radiation, the mechanism underlying the hyper radio-sensitivity has not yet been fully understood [12-14,18]. AT cells have no gross defect in DNA double strand break repair, however, several studies reported that a fraction of the initial DNA double strand breaks remained unrejoined in AT cells [19-23]. While most of the DNA double strand breaks are repaired by DNA-PK-dependent non-homologous end-joining (NHEJ), a subset of breaks, which are refractory to DNA repair, might require Artemis for processing [6,23,24]. As Artemis activity is regulated by phosphorylation by ATM [23,25-27], it was suggested that a lack of Artemis activity explains increased radiosensitivity of AT cells.

More recently, another possibility was proposed, in which ATM activity is required for reorganization of heterochromatin through phosphorylation of Kruppel-associated box-associated protein-1 (KAP1) [28]. This idea was based on the understanding that DNA damage foci in heterochromatin regions are more refractory to repair than those in euchromatin regions [29-32]. Mobilization of KAP-1 by ATM-dependent phosphorylation is necessary for foci removal from heterochromatin [33], suggesting that cells lacking ATM function accumulate residual DNA double strand breaks in heterochromatin regions. However, there was no direct evidence showing actual DNA double strand breaks persisted in heterochromatin. It was also reported that other ATM-independent mechanisms were involved in DNA repair in heterochromatin. For example, ATM-independent mobilization of HP1 from chromatin increased accessibility of DNA double strand breaks by repair factors [34]. Local chromatin relaxation in the vicinity of DNA double strand breaks was also mediated by ATP-dependent mechanism [29]. Thus, multiple pathways are involved in heterochromatic DNA repair. Therefore, it is still possible that increased radiosensitivity of AT cells does not solely stem from inability to repair DNA double strand breaks in heterochromatin [35].

Recently, cell cycle-dependent repair of DNA double strand breaks was examined in AT and Artemis-defective cells [22]. Since residual fractions of foci were similar between AT and Artemis-defective cells in G1, a subset of DNA double strand breaks seems to require processing by Artemis-dependent pathway. Therefore, we have asked whether any specific types of broken ends require ATM-dependent repair pathway. Here, we examined the repair kinetics of DNA double strand breaks in synchronized G1 cells treated with different restriction enzymes. Restriction endonucleases were introduced into cells by electroporation [36]. We found that ATM inhibition by KU55933 partially compromised repair of DNA double strand breaks created by *Pvu *II, *Rsa *I, *Eco *RI, and *Bam *HI, but not by *Pst *I and *Sph *I, indicating that ATM-dependent pathway is required for processing certain types of termini. Our results propose that a part of radiosensitivity in AT cells could be explained by defective repair of certain types of DNA double strand breaks induced by ionizing radiation.

## Results

### Induction of DNA damage foci by restriction endonuclease treatments

**Figure 1:**
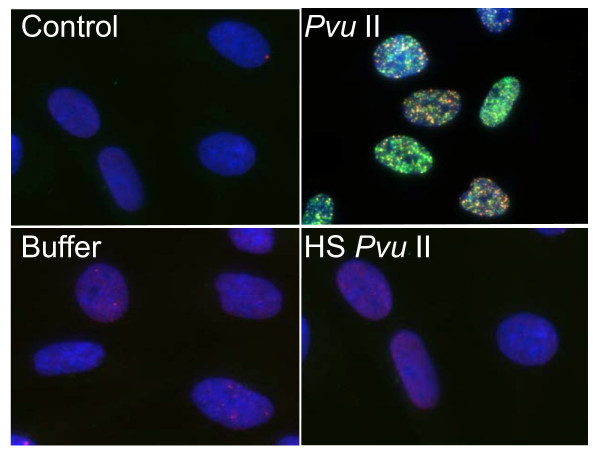
**Electroporation of cells with restriction endonucleases**. Synchronized normal human diploid cells in G1 were collected by trypsinization and suspended in PBS buffer. Enzyme reaction buffer and *Pvu *II (100 U) were mixed immediately before electroporation as described in METHODS. Cells were incubated for 2 hours before fixation. Control; no treatment, Buffer; cells were electroporated with reaction buffer, *Pvu *II; cells were treated with 100 U of *Pvu *II, HS *Pvu *II, cells were electroporated with *Pvu *II (100 U) heat inactivated at 80°C for 20 minutes.

Induction of DNA double strand breaks was examined by the foci formation of phosphorylated ATM and 53BP1. Because cells were electroporated in the presence of enzyme reaction buffer, we checked whether these conditions affected foci formation or not. As shown in Figure 1, electroporation of *Pvu *II induced phosphorylated ATM foci and 53BP1 foci, whereas no focus induction was observed in cells that underwent electroporation with buffer only. We also confirmed that the foci formation was dependent upon the enzyme activity, since cells electroporated with heat-inactivated *Pvu *II did not induce foci (HS *Pvu *II).

A variety of endonucleases were used in this study. Group I restriction endonucleases include *Pvu *II, *Rsa *I, *Bgl *I, *Eco *RV and *Sma *I, which create blunt ends. Group II enzymes including *Pst *I, *Sph *I, and *Kpn *I generate 3'-protruding ends. Group III enzymes include *Eco *RI, *Bam *HI, *Not *I, *Hind *III, and *Hinf *I, which produce 5'-protruding ends. While dose-dependent increase of foci-positive cells was observed, we decided to use 100 units as they were the optimum condition for the detection of the foci. Electroporation of cells with *Pvu *II, *Rsa *I, *Pst *I, *Sph *I and *Eco *RI induced foci in more than 80% of cells, while *Eco *RV, *Bam *HI and *Hinf *I could induce foci in approximately 50% of cells. In contrast, little or no foci was induced by 100 units of *Bgl *I, *Sma *I, *Kpn *I, *Not *I and *Hind *III, and no effect was observed even with increasing the amount of enzymes. Therefore, in the following experiments, we used six restriction endonucleases including *Pvu *II, *Rsa *I, *Pst *I, *Sph *I, *Eco *RI and *Bam *HI.

**Figure 2:**
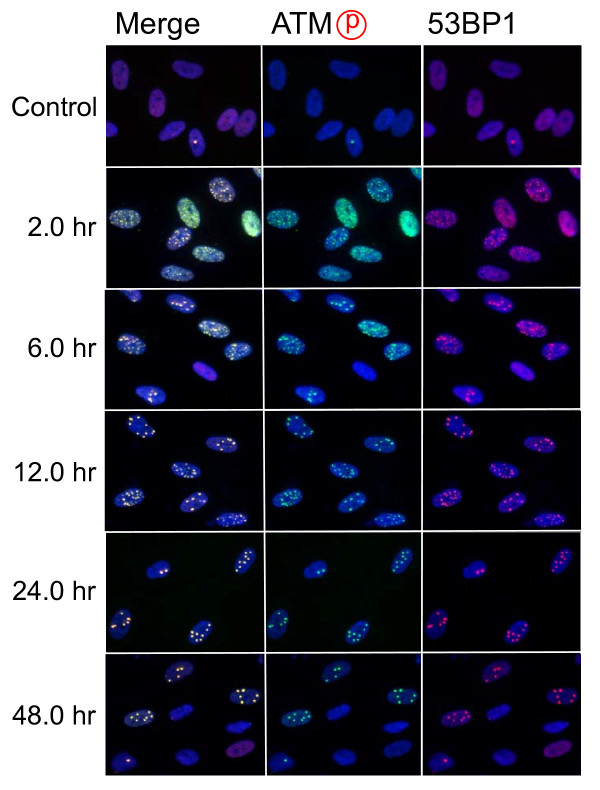
**Time-dependent foci formation by Pvu II**. Synchronized normal human diploid cells in G1 were electroporated with *Pvu *II (100 U) as described in METHODS. The cells were incubated for the time indicated before fixation.

**Figure 3:**
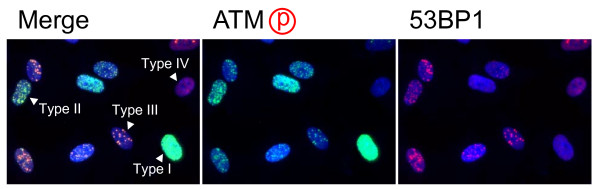
**Classification of the nuclei with foci**. Synchronized normal human diploid cells in G1 were electroporated with *Pvu *II (100 U) as described in METHODS, and they were incubated for 2 hours before fixation. The nuclei were classified into 4 types. Type I; nuclei with diffused phosphorylated ATM signal, but without 53 BP1 foci. Type II; nuclei with numerous foci more than 30 foci. Type III; nuclei with countable foci below 30. Type IV; nuclei without detectable foci.

After electroporation with restriction endonucleases, cells were incubated for 2 hours, which allow cells to attach on the coverslips. At this point, more than 90% of cells showed the signal of ATM phosphorylation (Figure 2). As shown in Figure 3, 30~40% of cells showed diffused foci signal throughout the nuclei, which were classified as Type I nuclei (Figure 3). Approximately 30% of cells had numerous foci, whose number was more than 30 (Type II nuclei), while 10~20% of cells contained countable numbers of foci (1~30)(Type III nuclei). Type IV nuclei were those without any foci. It should be noted that 53BP1 foci could not be detected in Type I nuclei. This is because 53BP1 is the protein recruited to the sites of phosphorylated ATM foci. Therefore, if multiple tiny foci of phosphorylated ATM were evenly distributed within the nucleus, 53BP1 might not be detected as the foci. In Type II and III nuclei, 53BP1 foci were always colocalized with phosphorylated ATM foci. Activated ATM transduces DNA damage signal through phosphorylation of the downstream effectors. In fact, we confirmed that phosphorylated ATM foci were also colocalized with phosphorylated 53BP1, phosphorylated histone H2AX, and phosphorylated NBS1 (See Additional file 1). In the subsequent study, we counted the number of 53BP1 foci colocalized with phosphorylated ATM foci.

### Repair of restriction endonuclease-induced foci

**Figure 4:**
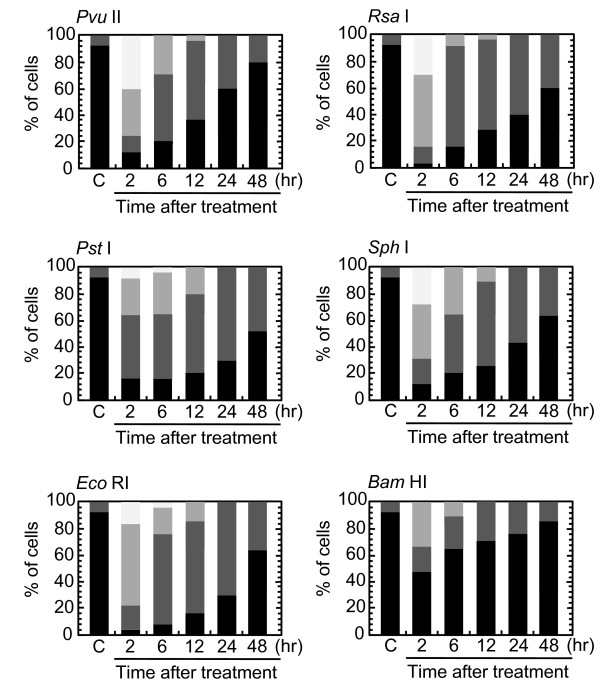
**Distribution of cells with different nuclear type**. Synchronized normal human diploid cells in G1 were electroporated with 100 U of various restriction endonucleases as described in METHODS. The cells were incubated for the time indicated before fixation. At least 1000 nuclei were examined. C; mock-treated control cells without restriction endonucleases. Black bars represent cells without foci (Type IV). Dark gray and light gray bars represent cells with Type III and Type II nuclei, respectively. Off-white bars represent cells with Type I nuclei.

Time-dependent decrease in the foci number was examined (Figure 2). After electroporation, at least one hour was needed to allow cells for firm attachment. Two hours after the treatment, the foci were already induced maximally, and more than 90% of cells were foci-positive after *Pvu *II-treatment (Figure 4). The percentage of Type I nuclei gradually disappeared thereafter, and more than 50% of cells lost foci within 24 hours after the treatment. By 48 hours after the treatment, more than 80% of cells repaired foci. Because the number of foci per nucleus was not uniformly distributed, the number of foci-negative cells might be overestimated by growth of the cells that were released from cell cycle arrest. Therefore, repair of foci was also assessed by the distribution of foci number per nucleus. As shown in Figure 3, the number of foci was also decreased with increasing times after the treatment. Between 6 and 24 hours after the treatment, the fraction of Type III nuclei seemed to be unchanged, as Type I and II nuclei were shifted to Type III nuclei, but the number of foci apparently decreased, indicating repair of DNA damage foci in Type III nuclei. Similar results were obtained in every cell treated with *Rsa *I, *Pst *I, *Sph *I, *Eco *RI and *Bam *HI (Figure 4).

### Effects of ATM inactivation on DNA damage foci repair

**Figure 5:**
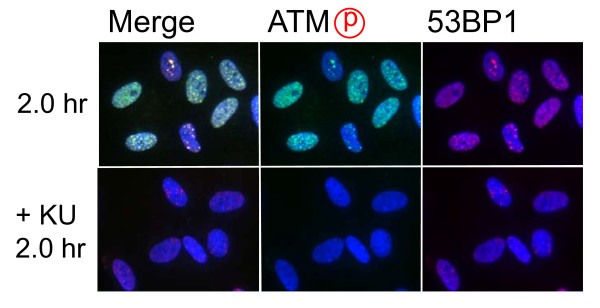
**Effect of ATM inhibitor on foci formation**. Synchronized normal human diploid cells in G1 were electroporated with *Pvu *II (100 U) as described in METHODS, and they were incubated for 2 hours before fixation. KU55933 (20 μM) was administrated 30 minutes before electroporation, and the cells were incubated with a medium containing KU55933 after electroporation.

**Figure 6:**
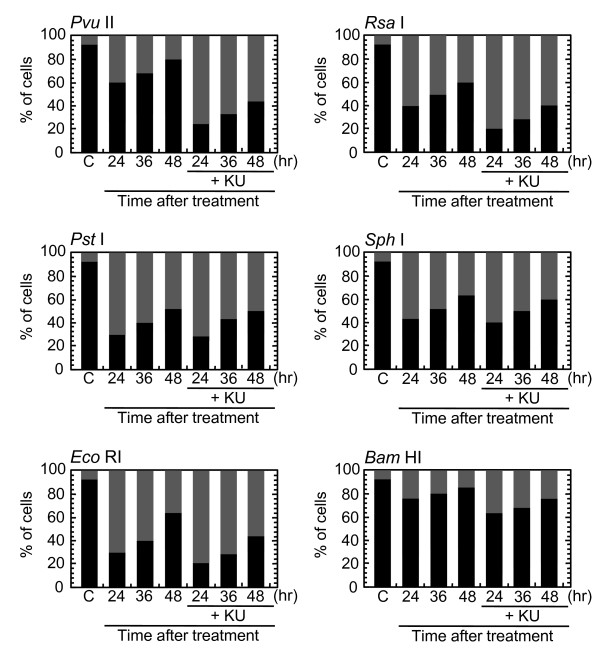
**Effect of ATM inhibition on DNA damage foci repair **Synchronized normal human diploid cells in G1 were electroporated with 100 U of various restriction endonucleases with or without 20 μM KU55933. The cells were incubated for the time indicated before fixation. At least 1000 nuclei were examined. C; mock-treated control cells without restriction endonucleases. Black bars and dark gray bars represent cells with Type IV and Type III nuclei, respectively.

**Figure 7:**
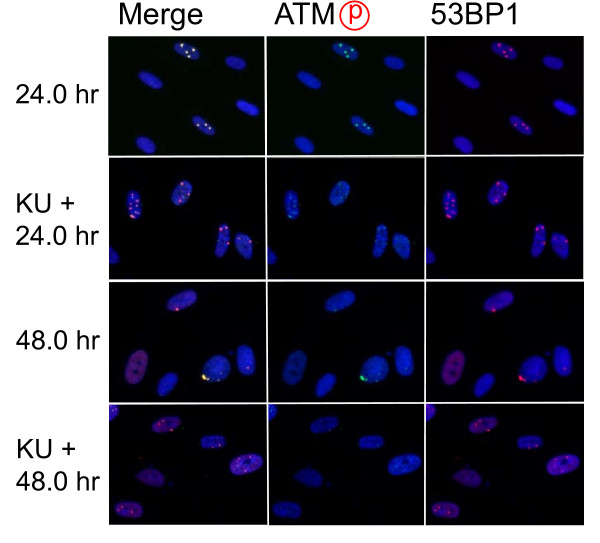
**Effect of ATM inhibition on distribution of foci number**. Synchronized normal human diploid cells in G1 were electroporated with 100 U of *Pvu *II with or without 20 μM KU55933. The cells were incubated for the time indicated before fixation.

**Figure 8:**
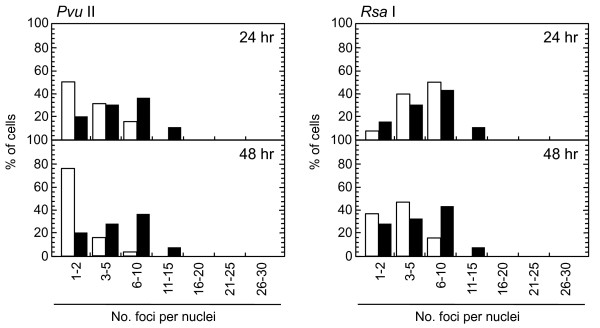
**Distribution of foci numbers per nucleus**. Synchronized normal human diploid cells in G1 were electroporated with 100 U of *Pvu *II and *Rsa *I with or without 20 μM KU55933. The cells were incubated for 24 or 48 hours after the treatment, and the foci numbers in Type III nuclei were counted. At least 500 nuclei were examined. Black bars: cells treated with KU55933. White bars: cells treated without KU55933.

**Figure 9:**
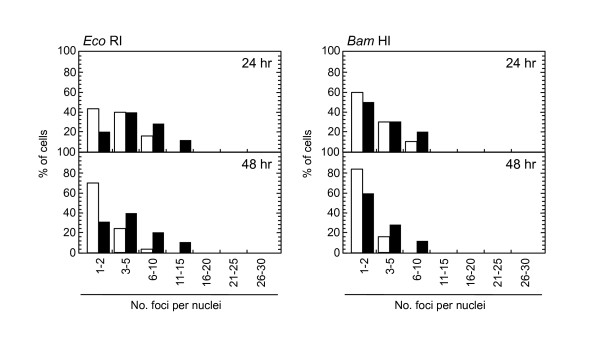
**Distribution of foci numbers per nucleus**. Synchronized normal human diploid cells in G1 were electroporated with 100 U of *Eco *RI and *Bam *HI with or without 20 μM KU55933. The cells were incubated for 24 or 48 hours after the treatment, and the foci numbers in Type III nuclei were counted. At least 500 nuclei were examined. Black bars: cells treated with KU55933. White bars: cells treated without KU55933.

**Figure 10:**
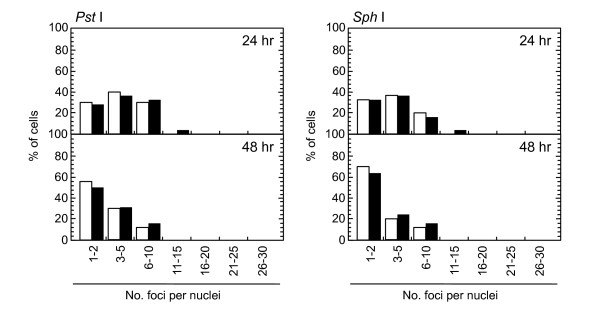
**Distribution of foci numbers per nucleus**. Synchronized normal human diploid cells in G1 were electroporated with 100 U of *Pst *I and *Sph *I with or without 20 μM KU55933. The cells were incubated for 24 or 48 hours after the treatment, and the foci numbers in Type III nuclei were counted. At least 500 nuclei were examined. Black bars: cells treated with KU55933. White bars: cells treated without KU55933.

Role of ATM-dependent repair pathway were examined by inhibiting ATM activity using an ATM specific inhibitor, KU55933. Suppression of ATM activity was checked by significant loss of phosphorylation of ATM at serine 1981 (Figure 5). Accordingly, foci formation of 53 BP1 was significantly compromised, although the effect was less profound compared with the suppressive effect on phosphorylated ATM foci. Because inhibition of ATM activity by KU55933 is reversible, the formation of phosphorylated ATM foci and 53 BP1 foci was visualized by incubating cells for 0.5 hour with a fresh medium without KU55933. The percentage of cells with Type III and IV nuclei was compared 24, 36, and 48 hours after the treatment. We confirmed that KU55933 treatment alone did not show any effect on the foci type distribution in the control cells. As shown in Figure 6, the increase of foci-negative nuclei was suppressed by KU55933 in cells treated with *Pvu *II, *Rsa *I, *Eco *RI and *Bam *HI, whereas, no such effect was observed in *Pst *I and *Sph *I-treated cells. The effect of KU55933 was more pronounced when the number of foci in Type III nuclei was compared (Figure 7). The distribution of foci number clearly showed an inhibitory effect of DNA damage foci repair by ATM inhibition in *Pvu *II, *Rsa *I, *Eco *RI, and *Bam *HI-treated cells (Figures 8 and 9). In contrast, the distribution of foci number does not show any significant difference in cells treated with *Pst *I and *Sph *I (Figure 10).

## Discussion

Use of restriction endonucleases to study the biological effects of DNA double strand breaks has been described for many years [36-38]. Previously, the formation of DNA double strand breaks was quantified by chromosome aberrations or by comet assay in restriction endonuclease-treated Chinese hamster cells [39-41] and human lymphoblastoid cells [37,38,40]. Here, we introduced various restriction endonucleases into G1-synchronized normal human fibroblast-like cells, and DNA double strand breaks were successfully detected by phosphorylated ATM foci and 53 BP1 foci. It is well established that the foci of DNA damage checkpoint factors can be used as reliable markers for DNA double strand breaks [42-44]. As phosphorylation of such factors was also induced in response to various stresses [45-47], we carefully determined whether electroporation by itself or the introduction of exogenous proteins did not cause phosphorylation of ATM. As shown in Figure 1, neither treatment with reaction buffer only nor electroporation with heat-inactivated *Pvu *II induced foci, indicating that foci formation exclusively depended upon the enzyme activity. Although various types of restriction endnucleases were examined in this study, not all of them were functional in normal human cells. The reason of this result was not known, but biochemical conditions including salt concentration might not be appropriate in the intact nuclei for some enzymes. According to the result shown in Figure 2, at least two-hour incubation after electroporation was sufficient for inducing DNA double strand breaks. Since the fraction of foci-negative cells was already increased slightly six hours after *Pvu *II treatment (Figure 4), the enzyme activity seemed to be active for the first few hours. Repair of DNA double strand breaks induced by restriction endonucleases was confirmed by the increase of the fraction of cells without foci (Type IV nuclei). It was also evidenced when the distribution of foci numbers in Type III nuclei was compared (Figures 8, 9 and 10).

Involvement of ATM-dependent pathway in foci repair was examined by inhibiting ATM activity by KU55933, which is a specific inhibitor for ATM [48]. As shown in Figures 5 and 7, KU55933 treatment significantly compromised phosphorylation of ATM, indicating that ATM activity was considerably inhibited. While the suppressive effect was less significant in 53 BP1 foci, it could be explained by phosphorylation-independent accumulation of 53 BP1, as described previously [49]. The increase in the fraction of Type IV nuclei was delayed by KU55933 in cells treated with *Pvu *II, *Rsa *I, *Eco *RI, and *Bam *HI. Although such inhibitory effects were not apparent at early times after the treatment (data not shown), noticeable effect was observed at later times (Figure 6). Similar result was reported in AT cells exposed to X-rays, in which no repair defect during 2 hours incubation after X-irradiation but the fraction of residual damage was significantly higher [22]. More striking effects were observed when the distribution of foci number was compared (Figures 8 and 9). Importantly, these inhibitory effects were not entirely detected in cells treated with *Pst *I and *Sph *I (Figures 6 and 10). Thus, these results indicate that a part of DNA double strand breaks, created by restriction endonucleases generating blunt ends and 5'-protruding ends, requires ATM-dependent DNA repair pathway.

The major pathway responsible for repair of DNA double strand breaks in G1 is DNA-PK-dependent NHEJ [1-6], and our results and others indicated that NHEJ pathway could repair most of the restriction endonuclease-induced DNA double strand breaks irrespective of ATM deficiency [37,38]. However, ATM inhibition partly compromised repair of foci, especially, those persisted foci for over 24 hours. According to the previous results, these residual foci possibly represented chromatins with unreparable DNA breaks, DNA breaks refractory for repair, and those harboring mis-rejoined DNA damage [7,50]. Since a part of residual foci gradually reduced in number in the presence of ATM activity, it seems likely that they represent DNA breaks refractory for repair by a conventional NHEJ pathway. We presumed that such slowly-repairing DNA damage required ATM-dependent pathway. The important information was that ATM activity was required for the repair of 5'-protruding and blunt ends, whereas it did not show any role in repair of 3'-protruding DNA double strand breaks. Therefore, it is likely that a part of 5'-protruding and blunt termini requires ATM activity to expose 3'-protruding ends, whose process needs 5' to 3' exonuclease activity.

Then, how does ATM activity regulate end processing? One possibility is that Artemis is involved in this processing. Artemis was the first component, involved in NHEJ pathway, which is phosphorylated by ATM [23,25]. An epistasis-type analysis demonstrated that AT cells and Artemis-defective cells showed identical DNA repair phenotypes [22]. Furthermore, addition of ATM inhibitor to Artemis-defective cells resulted in no additive effect on repair of residual damage. Thus, it was concluded that ATM and Artemis function in the same DNA repair pathway. Artemis has 5' to 3' exonuclease activity towards single stranded DNA, while it also acquires endonuclease activity in the presence of DNA-PK [25,51,52]. Although subsequent studies have demonstrated that DNA-PK is an essential factor for Artemis activity [51], ATM-dependent phosphorylation was suggested to inhibit regulation of Artemis by DNA-PK-dependent phosphorylation [53]. Therefore, it is possible that ATM regulates exonuclease activity of Artemis involved in end-processing of broken DNA ends. Although we need further investigation, a plausible mechanism is that some residual DNA breaks need processing by Artemis to create the 3'-protruding ends. According to the recent review, the initial step of DNA-PK-dependent NHEJ starts by binding of Ku80/70 heterodimers to the broken ends [6]. In most cases, DNA-PKcs tethers the broken ends by interacting with Ku80/70 heterodimers. But, clustered damage was introduced by restriction endonucleases in a localized area, DNA-PK-dependent pathway can not be functional anymore, and backup repair system takes place. Or, some broken termini might be attacked by endogenous nucleases, which results in incompatible ends. In either case, DNA ends may need processing by Artemis.

Although ATM activity is required for reorganizing heterochromatin through KAP-1 phosphorylation [28], it might not explain the results obtained in this study. If ATM-dependent heterochromatin reorganization was involved in repair of residual foci, ATM inhibition affected repair of residual foci irrespective of the structure of broken ends. However, this assumption was not in agreement with the results, in which the repair of *Pst *I- and *Sph *I-induced damage was insensitive to ATM inhibition. Thus, it is more likely that ATM activity plays a role in activating Artemis-dependent DNA processing a subset of DNA double strand breaks. Although future studies should define the molecular nature of this process, our results suggest that hyper radiosensitivity of AT cells might be explained in part by a defect in this process.

## Conclusions

Radiosensitive AT cells showed difficulty to rejoin a small fraction of DNA double strand breaks. Our current study demonstrated that a part of residual blunt and 5'-protruding ends required ATM activity, but repair of residual 3'-protruding ends was not affected by ATM inhibition. Thus, it is concluded that the defect in ATM-dependent DNA repair pathway, which is indispensable for the repair of subsets of residual breaks, could be a cause of increased radiosensitivity of AT cells.

## Methods

### Cell culture

Normal human diploid fibroblast-like cells [54,55], which derived from embryonic dermal tissue, were cultured in MEM supplemented with 10% fetal bovine serum (TRACE Bioscience PTY Ltd., Australia). To obtain synchronized cells the cells were subcultured at a high density for days with changing medium every 3 days. After 7 days-synchronization, more than 95% of cells were in G0/G1. The ATM kinase activity was inhibited by a specific inhibitor, KU55933, and 20 μM of KU55933 was administrated 30 min before electroporation. Immediately after electroporation, a fresh medium containing 20 μM of KU55933 was fed, and the cells were cultured at 37°C in a 5% CO_2 _incubator until they were fixed. In order to visualize phosphorylated ATM foci and 53 BP1 foci, the cells were incubated for one with a fresh medium without KU55933.

### Introduction of restriction endonucleases by electroporation

Synchronized cells were washed with phosphate-buffered saline (PBS) twice, collected by trypsinization and resuspended in PBS at a concentration of 2 × 10^6^/ml. Then, 450 μl of cell suspension was mixed with 50 μl of reaction buffer, and restriction endonucleases were added before electroporation (pulse height and width were 400 V/cm and 1000 msec, respectively). Immediately after electroporation, a fresh medium was fed, and cells were plated onto sterilized 22 × 22 mm cover slips at a density of 5 × 10^4 ^cells per slip. The cells were incubated at 37°C in a 5% CO_2 _incubator until they were fixed. Restriction endonucleases were obtained from Nippon Gene (Tokyo, Japan).

### Immunofluorescence

Cells cultured on coverslips were fixed with 4% formaldehyde for 10 min, permeabilized with 0.5% Triton X-100 for 5 min, and were washed extensively with phosphate-buffered saline (PBS). Fixation and permeabilization were performed on ice. The primary antibodies were diluted in 100 μl of TBS-DT (20 mM Tris-HCl, pH7.6, 137 mM NaCl, containing 50 mg/ml skim milk and 0.1% Tween-20), and the antibodies were applied on the coverslips. The samples were incubated for 2 hours in a humidified CO_2 _incubator at 37°C. Then, the primary antibodies were washed with PBS, and Alexa488-labelled anti-mouse or Alexa594-labelled anti-rabbit IgG antibodies (Molecular Probes, Inc., OR) were added. The coverslips were incubated for 1 hour in a humidified CO_2 _incubator at 37°C, washed with PBS, and counterstained with 0.1 mg/ml of DAPI. The samples were examined with a F3000B fluorescence microscope (Leica, Tokyo). Digital images were captured and the images were analyzed by FW4000 software (Leica, Tokyo). In order to quantify the fluorescence intensity, green dot-like signals were marked, and the sum of the pixel intensity within the marked area was calculated by FW4000 software. The primary antibodies used in this study were mouse anti-phosphorylated ATM at serine 1981 monoclonal antibody (Clone 10 H11.E12, Rockland, Gilbertsville, PA), rabbit anti-53 BP1 polyclonal antibody (A300-272 A, BETHYL, Montgomery, TX), rabbit anti-phosphorylated NBS1 at serine 343 polyclonal antibody (A300-189 A, BETHYL, Montgomery, TX), rabbit anti-phosphorylated histone H2AX at serine 139 polyclonal antibody (A300-081 A, BETHYL, Montgomery, TX), and rabbit anti-phosphorylated 53 BP1 at serine 1778 polyclonal antibody (2675, Cell Signaling Technology Japan, Tokyo).

## Competing interests

The authors declare that they have no competing interests.

## Authors' contributions

KS conceived of the study, carried out the immunoflorescence study, and drafted the manuscript. MT carried out the immunoflorescence study and performed the statistical analysis. YO participated in the design of the study. MY participated in the design of the study and carried out the immunoflorescence study. MS participated in the design of the study. SY helped to draft the manuscript. All authors read and approved the final manuscript.

## Supplementary Material

Additional file 1 **Colocalization of the foci of phosphorylated proteins**. Synchronized normal human diploid cells in G1 were electroporated with *Pvu *II (100 U) as described in METHODS. The cells were incubated for 12 hours before fixation. Click here for file

## References

[B1] Burma S Chen BP Chen DJ Role of non-homologous end joining (NHEJ) in maintaining genomic integrity DNA Repair 2006 5 1042 1048 10.1016/j.dnarep.2006.05.026 16822724

[B2] Sonoda E Hochegger H Saberi A Taniguchi Y Takeda S Differential usage of non-homologous end-joining and homologous recombination in double strand break repair DNA Repair 2006 5 1021 1029 10.1016/j.dnarep.2006.05.022 16807135

[B3] Wyman C Kanaar R DNA double-strand break repair: all's well that ends well Annu Rev Genet 2006 40 363 383 10.1146/annurev.genet.40.110405.090451 16895466

[B4] Jeggo P Lavin MF Cellular radiosensitivity: How much better do we understand it? Int J Radiat Biol 2009 85 1061 1081 10.3109/09553000903261263 19995233

[B5] Lisby M Rothstein R Choreorgaphy of recombination proteins during the DNA damage response DNA Repair 2009 8 1068 1076 10.1016/j.dnarep.2009.04.007 19473884 PMC2729071

[B6] Mahaney BL Meek K Lees-Miller SP Repair of ionizing radiation-induced DNA double-strand breaks by non-homologous end-joining Biochem J 2009 417 639 650 10.1042/BJ20080413 19133841 PMC2975036

[B7] Agarwal S Tafel AA Kanaar R DNA double-strand break repair and chromosome translocations DNA Repair 2006 5 1075 1081 10.1016/j.dnarep.2006.05.029 16798112

[B8] Jeggo P Lobrich M Contribution of DNA repair and cell cycle checkpoint arrest to the maintainance of genomic stability DNA Repair 2006 5 1192 1198 10.1016/j.dnarep.2006.05.011 16797253

[B9] Weinstock DM Brunet E Jasin M Induction of chromosomal translocations in mouse and human cells using site-specific endonucleases J Natl Cancer Inst Monogr 2008 39 20 24 10.1093/jncimonographs/lgn009 18647997 PMC3261771

[B10] Bartek J Bartkova J Lukas J DNA damage signalling guards against activated oncogenes and tumour progression Oncogene 2007 26 7773 7779 10.1038/sj.onc.1210881 18066090

[B11] Harper JW Elledge SJ The DNA damage response: ten years after Mol Cell 2007 28 739 745 10.1016/j.molcel.2007.11.015 18082599

[B12] Shiloh Y ATM and related proteins kinases: safeguarding genome integrity Nat Rev Cancer 2003 3 155 168 10.1038/nrc1011 12612651

[B13] Kitagawa R Kastan MB The ATM-dependent DNA damage signaling pathway Cold Spring Harb Symp Quant Biol 2005 70 99 109 10.1101/sqb.2005.70.002 16869743

[B14] Lavin MF Ataxia-telangiectasia: from a rare disorder to a paradigm for cell signalling and cancer Nat Rev Mol Cell Biol 2008 9 759 769 10.1038/nrm2514 18813293

[B15] Bakkenist CJ Kastan MB DNA damage activates ATM through intermolecular autophosphorylation and dimer dissociation Nature 2003 421 499 506 10.1038/nature01368 12556884

[B16] Jackson SP Bartek J The DNA-damage response in human biology and disease Nature 2009 461 1071 1078 10.1038/nature08467 19847258 PMC2906700

[B17] Jackson SP The DNA-damage response: new molecular insights and new approaches to cancer therapy Biochem Soc Trans 2009 37 483 494 10.1042/BST0370483 19442242 PMC2907489

[B18] Pollard JM Gatti RA Clinical radiation sensitivity with DNA repair disorders: an overview Int J Radiat Oncol Biol Phys 2009 74 1323 1331 19616740 10.1016/j.ijrobp.2009.02.057 PMC2725446

[B19] Foray N Priestley A Alsbeih G Badie C Capulas EP Arlett CF Malaise EP Hypersensitivity of ataxia-telangiectasia fibroblasts to ionizing radiation is associated with a repair deficiency of DNA double-strand breaks Int J Radiat Biol 1997 72 271 283 10.1080/095530097143266 9298107

[B20] Jeggo PA Carr AM Lehmann AR Splitting the ATM: distinct repair and checkpoint defects in ataxia-telangiectasia Trends Genet 1998 14 312 316 10.1016/S0168-9525(98)01511-X 9724963

[B21] Kuhne M Riballo E Rief M Rothkamm K Jeggo PA Lobrich M A double-strand break repair defect in ATM-deficient cells contributes to radiosensitivity Cancer Res 2004 64 500 508 10.1158/0008-5472.CAN-03-2384 14744762

[B22] Riballo E Kuhne M Rief N Doherty A Smith GCM Recio MJ Reis C Dahm K Fricke A Krempler A Parker AR Jackson SP Gennery A Jeggo PA Lobrich M A pathway of double-strand break rejoining dependent upon ATM, Artemis, and proteins locating to γ-H2AX foci Mol Cell 2004 16 715 724 10.1016/j.molcel.2004.10.029 15574327

[B23] Jeggo PA Lobrich M Artemis links ATM to double strand break rejoining Cell Cycle 2005 4 359 362 15684609 10.4161/cc.4.3.1527

[B24] Lobrich M Jeggo PA Harmonising the response to DSBs: a new string in the ATM bow DNA Repair 2005 4 749 759 10.1016/j.dnarep.2004.12.008 15978533

[B25] Dahm K Functions and regulation of human Artemis in double strand break repair J Cell Biochem 2007 100 1346 1351 10.1002/jcb.21226 17211852

[B26] Poinsignon C de Chasseval R Soubeyrand S Moshous D Fischer A Hache RJG de Villartay JP Phosphorylation of Artemis following irradiation-induced DNA damage Eur J Immunol 2004 34 3146 3155 10.1002/eji.200425455 15468306

[B27] Zhang X Succi J Feng Z Prithivirasingh S Story MD Legersky R Artemis is a phosphorylation target of ATM and ATR and is involved in the G2/M DNA damage checkpoint response Mol Cell Biol 2004 24 9207 9220 10.1128/MCB.24.20.9207-9220.2004 15456891 PMC517881

[B28] Goodarzi AA Noon AT Deckbar D Ziv Y Shiloh Y Lobrich M Jeggo PA ATM signaling facilitates repair of DNA double-strand breaks associated with heterochromatin Mol Cell 2008 31 167 177 10.1016/j.molcel.2008.05.017 18657500

[B29] Kruhlak MJ Celeste A Dellaire G Fernadez-Capetillo O Muller WG McNally JG Bazett-Jones DP Nessenzweig A Changes in chromatin structure and mobility in living cells at sites of DNA double-strand breaks J Cell Biol 2006 172 823 834 10.1083/jcb.200510015 16520385 PMC2063727

[B30] Ball AR Yokomori K Revisiting the role of heterochromatin protein 1 in DNA repair J Cell Biol 2009 185 573 575 10.1083/jcb.200904033 19451270 PMC2711565

[B31] Cowell IG Sunter NJ Singh PB Austin CA Durkacz BW Tilby MJ Gamma-H2AX foci form preferentially in euchromatin after ionising-radiation PLoS ONE 2007 2 e1057 10.1371/journal.pone.0001057 10.1371/journal.pone.0001057 17957241 PMC2020439

[B32] Murga M Jaco I Fan Y Soria R Martinez-Pastor B Cuadrado M Yang SM Blasco MA Skoultchi AI Fernandez-Capetillo O Global chromatin compaction limits the strength of the DNA damage response J Cell Biol 2007 178 1101 1108 10.1083/jcb.200704140 17893239 PMC2064646

[B33] Goodarzi AA Noon AT Jeggo PA The impact of heterochromatin on DSB repair Biochem Soc Trans 2009 37 569 576 10.1042/BST0370569 19442252

[B34] Ayoub N Jeyasekharan AD Bernal JA Venkitaraman AR HP1-β mobilization promotes chromatin changes that initiate the DNA damage response Nature 2008 453 682 686 10.1038/nature06875 18438399

[B35] Fernadez-Capetillo O Nussenzweig A ATM breaks into heterochromatin Mol Cell 2008 31 303 304 10.1016/j.molcel.2008.07.004 18691960

[B36] Winegar RA Phillips JW Youngblom JH Morgan WF Cell electroporation is a highly efficient method for introducing restriction endonucleases into cells Mutat Res 1989 225 49 53 10.1016/0165-7992(89)90032-8 2536473

[B37] Liu N Bryant PE Response of ataxia telangiectasia cells to restriction endonuclease induced DNA double-strand breaks: I. Cytogenetic characterization Mutagenesis 1993 8 503 510 10.1093/mutage/8.6.503 8133779

[B38] Liu N Bryant PE Enhanced chromosomal response of ataxia-telangiectasia cells to specific types of DNA double-strand breaks Int J Radiat Biol 1994 66 Supple 6 115 121 10.1080/09553009414551941 7836838

[B39] Bryant PE Use of restriction endonucleases to study relationships between DNA double-strand breaks, chromosomal aberrations and other end-points in mammalian cells Int J Radiat Biol 1988 54 869 890 10.1080/09553008814552291 2903886

[B40] Bryant PE Johnston PJ Restriction-endonuclease-induced DNA double-strand breaks and chromosomal aberrations in mammalian cells Mutat Res 1993 299 289 296 10.1016/0165-1218(93)90105-M 7683096

[B41] Ortiz T Pinero J Cortes F Chromosome damage induced by combined treatments with restriction endonucleases introduced into CHO cells by single or double electroporation Mutat Res 1995 327 161 169 7870084 10.1016/0027-5107(94)00183-6

[B42] Rogakou EP Boon C Redon C Bonner WM Megabase chromatin domains involved in DNA double-strand breaks in vivo J Cell Biol 1999 146 905 915 10.1083/jcb.146.5.905 10477747 PMC2169482

[B43] Olive PL Detection of DNA damage in individual cells by analysis of histone H2AX phosphorylation Methods Cell Biol 2004 75 355 373 full_text 15603433 10.1016/s0091-679x(04)75014-1

[B44] FitzGerald JE Grenon M Lowndes NF 53 BP1: function and mechanism of focal recruitment Biochem Soc Trans 2009 37 897 904 10.1042/BST0370897 19614615

[B45] Fernandez-Capetillo O Lee A Nussenzweig M Nussenzweig A H2AX: the histone guardian of the genome DNA Repair 2004 3 959 967 10.1016/j.dnarep.2004.03.024 15279782

[B46] Bonner WM Redon CE Dickey JS Nakamura AJ Sedelnikova OA Solier S Pommier Y GammaH2AX and cancer Nat Rev Cancer 2008 8 957 967 10.1038/nrc2523 19005492 PMC3094856

[B47] Kinner A Wu W Staudt C Iliakis G Gamma-H2AX in recognition and signaling of DNA double-strand breaks in the context of chromatin Nucleic Acids Res 2008 36 5678 5694 10.1093/nar/gkn550 18772227 PMC2553572

[B48] Hickson I Zhao Y Richardson CJ Green SJ Martin NM Orr AI Reaper PM Jackson SP Curtin NJ Smith GC Identification and characterization of a novel and specific inhibitor of the ataxia-telangiectasia mutated kinase ATM Cancer Res 2004 64 9152 9159 10.1158/0008-5472.CAN-04-2727 15604286

[B49] Huyen Y Zgheib O Ditullio RA Jr Gorgoulis VG Zacharatos P Petty TJ Sheston EA Mellert HS Stavridi ES Halazonetis TD Methylated lysine 79 of histone H3 targets 53 BP1 to DNA double-strand breaks Nature 2004 432 406 411 10.1038/nature03114 15525939

[B50] Kato TA Okayasu R Bedford JS Signatures of DNA double strand breaks produced in irradiated G1 and G2 cells persist into mitosis J Cell Physiol 2009 219 760 765 10.1002/jcp.21726 19206160

[B51] Goodarzi AA Yu Y Riballo E Douglas P Walker SA Ye R Harer C Marchetti C Morrice N Jeggo PA Lees-Miller SP DNA-PK autophosphorylation facilitates Artemis endonuclease activity The EMBO J 2006 25 3880 3889 10.1038/sj.emboj.7601255 PMC1553186 16874298

[B52] Yannone SM Khan IS Zhou RZ Zhou T Valerie K Povirk LF Coordinate 5' and 3' endonucleolytic trimming of terminally blocked blunt DNA double-strand break ends by Artemis nuclease and DNA-dependent protein kinase Nucleic Acids Res 2008 36 3354 3365 10.1093/nar/gkn205 18440975 PMC2425473

[B53] Beucher A Birraux J Tchouandong L Barton O Shibata A Conrad S Goodarzi AA Krempler A Jeggo PA Lobrich M ATM and Artemis promote homologous recombination of radiation-induced DNA double-strand breaks in G2 The EMBO J 2009 28 3413 3427 10.1038/emboj.2009.276 PMC2752027 19779458

[B54] Suzuki K Okada H Yamauchi M Oka Y Kodama S Watanabe M Qualitative and quantitative analysis of phosphorylated ATM foci induced by low dose ionizing radiation Radiat Res 2006 165 499 504 10.1667/RR3542.1 16669703

[B55] Watanabe M Suzuki M Suzuki K Nakano K Watanabe K Effect of multiple irradiation with low doses of gamma-rays on morphological transformation and growth ability of human embryo cells in vitro Int J Radiat Biol 1992 62 711 718 10.1080/09553009214552661 1362764

