# Supplementary figures and images for: Requirement of ATM-dependent pathway for the repair of a subset of DNA double strand breaks created by restriction endonucleases
Source: Genome Integr. 2010 May 26;1:4. doi: 10.1186/2041-9414-1-4 (PMC2907562; doi:10.1186/2041-9414-1-4)

## Slide 1
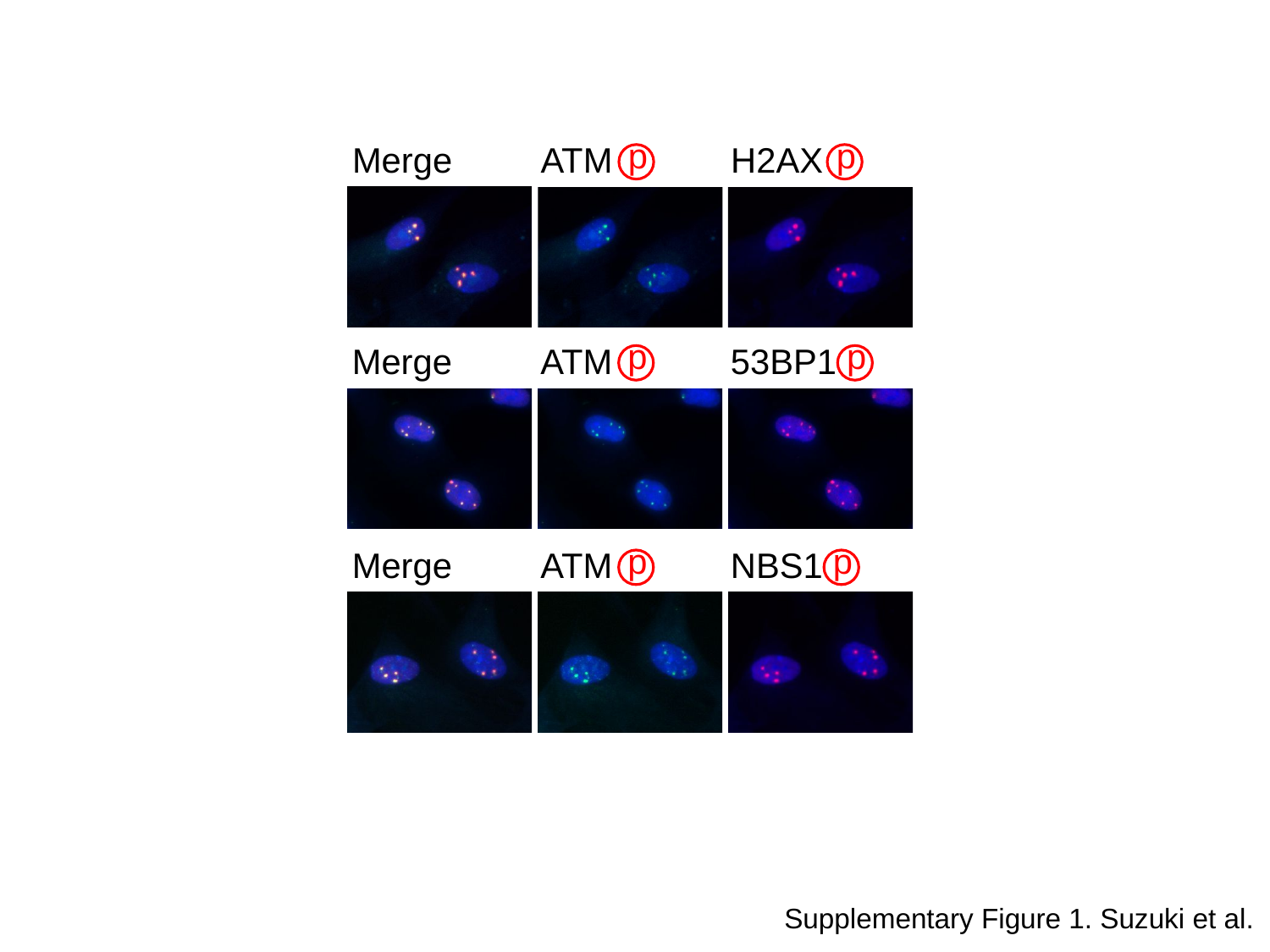

p
p
Merge
ATM
H2AX
p
p
Merge
ATM
53BP1
p
p
Merge
ATM
NBS1
Supplementary Figure 1. Suzuki et al.

Supplement: Additional file 1 — Colocalization of the foci of phosphorylated proteins. Synchronized normal human diploid cells in G1 were electroporated with Pvu II (100 U) as described in METHODS. The cells were incubated for 12 hours before fixation. [file 2041-9414-1-4-S1.PPT]
